# Supplementary material for: Modified in-vitro AATCC-100 procedure to measure viable bacteria from wound dressings
Source: PLoS One. 2024 Mar 21;19(3):e0298829. doi: 10.1371/journal.pone.0298829 (PMC10956864; doi:10.1371/journal.pone.0298829)
Supplement: Attachment — Submitted filename: Response to Reviewers.docx [file pone.0298829.s002.docx]

Dear *PLOS ONE* editorial and Review Members,

RE: PONE-D-23-17876/RESPONSE TO REVIEWERS.

Thank you, for the suggestions to improve our manuscript – please find our responses to the reviewers’ suggestions, comments, and recommended areas of improvement. We have highlighted in bold the reviewer’s comments and our statements are provided below each recommendation.

We value these insights and would welcome review of these responses and to our updated manuscript, (**Modified in-vitro AATCC-100 procedure to measure viable bacteria from wound dressings**; PONE-D-23-17876).

Thank you for your care

Sincerely,

Sang Hyuk Lee Ph. D

[sleejhu361@gmail.com /](mailto:sleejhu361@gmail.com%20/) sang.lee2@fda.hhs.gov

**PLOS style, financial disclosure, FDA acronym, Acknowledgment section, Table 1:**

We updated the cover letter and manuscript layout according to the guidelines. Please, find these changes in the corresponding sections of the manuscript.

**Reviewer 1: Introduction, Methods, Statistical sections rearrangement, Methods reference addition**

We updated each of the sections highlighted by the reviewers, accordingly—please, see each section for the described rearrangement of information.

**Reviewer 1: Low resolution pictures**

We exported the figures again at a higher resolution, please find these figures with this submission.

**Reviewer 2: Specific comments; Title, abstract elaboration, continuous introduction, reformatted methods**

We appreciate the recommendations provided by the reviewers, regarding the respectful details requested for the abstract, introduction, and modifications to the methods section—these are addressed in the revised manuscript.

**Reviewer 2: Specific comments; clarification of positive and negative controls**

We replaced the term previously used as “positive and negative treatment” with “positive and negative control” to better illustrate our findings.

**Reviewer 2: Fig S7: CFU / mL to Log CFU / mL**

The purpose of the bacteria growth characterization curve is not only to show the expected logarithmic bacteria growth pattern but also to fit to the similarly logarithmic time vs OD_600_ graph to make a calibration curve with high R squared value (supp fig 7 and 8). A time vs log CFU / mL may present a linear trend, yet it would distort the calibration curves thereof.

**Reviewer 2: Fig S7: statistical analysis**

Thank you for your valuable feedback. Regarding the calibration curve in Fig S7, which uses the abline fitting, the intention is to depict the relationship between two variables represented by discrete data points. We understand the importance of selecting appropriate statistical analyses, and in this context, ANOVA might not be the most suitable choice given it evaluates differences in means across multiple groups. Instead, for this specific analysis, we relied on the standard deviation from the 24h incubation combined with the plate-count method to provide a more precise assessment. We have also revisited the log reduction analysis of microbial viability/culturability, ensuring that we use a suitable statistical approach. We hope this clarifies our methodology.

**Reviewer 2: Statistical analysis of all figures**

1. **Analysis Approach** We concur with your suggestion regarding the selection of statistical tests. Given our intention to compare gene expressions across both the ‘method’ and ‘day’ factors in P. aeruginosa and S. aureus respectively, we employed a two-way ANOVA.
2. **Post-hoc pairwise Comparisons Analysis**: We'd like to clarify that we had already conducted a post-hoc pairwise comparisons analysis following the two-way ANOVA using the Tukey's HSD test. This was incorporated in the original version to ascertain specific differences among the levels of the factors. We have ensured that this is emphasized more clearly in our revised manuscript.
3. **Clarification on Statistical Analysis**: To ensure clarity and consistency in our statistical analyses, for any other figures presenting more than one independent factor, we have employed the two-way ANOVA approach and subsequently performed a post-hoc pairwise comparisons analysis to account for multiple comparisons.
4. **Updates to Figures and Legends:** In response to your feedback, we have updated the figures and legends. The plots have been revised with increased resolution, and we have updated the markers of statistical significance to ensure clarity.

**Reviewer 2: Additional recent references**

We updated the manuscript accordingly with references suggested by the reviewer and appreciate the recommendations.

**Reviewer 2: Our response regarding additional in-vivo analysis**

We appreciate the significance of the experiments recommended by the reviewers regarding in vivo testing and silver ion release measurements from the wound dressings. These experiments would benefit the scope of this work in the future to enhance our knowledge of product efficacy. To provide clarity on the present work, we would appreciate sharing the following:

At present, regulatory review and approval of ion-containing wound dressings to test anti-microbial properties for regulatory ‘indications of use’ in the U.S. require in vitro testing using the standard AATCC-100. Uniquely, the goal of this work was to compare the regulatory standard accepted by FDA, AATCC-100 for microbial viability testing with methods, such as qRT-PCR which can capture viable but non-culturable organisms (dormant organisms, though viable, which may not actively grow using agarose plating methods).  Molecular methods (such as qRT-PCR) allow us to measure non-culturable organisms, that otherwise could be considered non-viable if measured using plating techniques as a sole method. Using qRT-PCR we measured for gene transcription (mRNA synthesis) specific to 16sRNA – mRNA to this gene is produced by viable bacteria and can be used to measure for small concentrations of organisms that may not be detectable through serial dilution and plating. Therefore, the focus of our work was markedly to compare methods of detection for organisms exposed to silver ion from the wound dressings.

Related to this point of methods comparison, the current standard has a 24h test period—however some metal-ion containing wound dressings may indicate longer periods of use.  Therefore, the second goal of this work was to measure microbial viability at time points greater than 24h, to measure survival or proliferation of surviving bacteria. Additionally, this test provided us with the opportunity to examine if agar-plating methods captured culturable organisms and compare these results with viable organisms captured using qRT-PCR.  Again, this provides us with the opportunity to test currently regulatory standards (specifically in this case the period of incubation and testing (24h)) with longer test periods.

The measurement of silver ion will be a valuable test to incorporate into future iterations of this research. At present, this study focused on testing these methods that could help address products that contain antimicrobial metal ions, in addition to Ag, this includes Au, Cu, Zn, Fe, and Ti [1]. In future studies in addition to Ag concentrations we may measure other ions over extended test periods to identify what the levels of ions are within the dressing. At present, this study, though we tested Ag ions, is not uniquely focused on silver-ion containing dressings but is used as an example test product to assist with evaluating our microbial viability test method, using qRT-PCR.

Similarly, we value the experimental data and work by Lakshimipriya Sethuram et al [2], which describes how these products perform on a systematic level that includes biology important to the immune system (innate and adaptive), the contributions of other skin organisms, and inter/intra kingdom communication that contribute to a dynamic wound environment for product testing. As our goal was to evaluate current standards used specifically for in vitro testing, for regulatory review and product clearance, at this time our work is not focused on animal testing as it would be external to the scope of our regulatory science research. We value the insights through academia and other research bodies to help with our collective understanding of product efficacy and provided through.

1.	Wang, H., et al., *Application of metal-based biomaterials in wound repair.* Engineered Regeneration, 2021. **2**: p. 137-153.

2.	Sethuram, L., et al., *Effects and formulation of silver nanoscaffolds on cytotoxicity dependent ion release kinetics towards enhanced excision wound healing patterns in Wistar albino rats.* RSC Adv, 2019. **9**(61): p. 35677-35694.
