# Supplementary material for: Modified in-vitro AATCC-100 procedure to measure viable bacteria from wound dressings
Source: PLoS One. 2024 Mar 21;19(3):e0298829. doi: 10.1371/journal.pone.0298829 (PMC10956864; doi:10.1371/journal.pone.0298829)
Supplement: Attachment — Submitted filename: Response to Reviewers.docx [file pone.0298829.s003.docx]

Dear *PLOS ONE* editorial and Review Members,

RE: PONE-D-23-17876/RESPONSE TO REVIEWERS.

Thank you, for the suggestions to improve our manuscript – please find our responses to the reviewers’ suggestions, comments, and recommended areas of improvement. We have highlighted in bold the reviewer’s comments point by point and our statements are provided below each recommendation.

We value these insights and would welcome review of these responses and to our updated manuscript, (**Modified in-vitro AATCC-100 procedure to measure viable bacteria from wound dressings**; PONE-D-23-17876).

Thank you for your care

Sincerely,

Sang Hyuk Lee Ph. D

[sleejhu361@gmail.com /](mailto:sleejhu361@gmail.com%20/) sang.lee2@fda.hhs.gov

**PLOS style financial disclosure:**

We appreciate the assistance from Solna Carreon Santos who kindly helped us meet financial disclosure information to meet PLOS style. Please let us know if other specific statements are needful.

**Reviewer 1:**

1. **The introduction section needs to be summarized and rearranged again**

We updated the introduction by summarizing and rearranging details in increasing technicality.

2. **The methods section you didn't mention any reference even many of the methods are already known. Beside that you don't need to mention the full method in details if it is known just mention the reference.**

The original method of AATCC – 100 is documented in the literature and referenced:

Wiegand, C., et al., *In vitro assessment of the antimicrobial activity of wound dressings: influence of the test method selected and impact of the pH.* J Mater Sci Mater Med, 2015. **26**(1): p. 5343.

3. **The figures should be provided in more clear form as the resolution is very bad.**

We exported the figures again at a higher resolution, please find these figures with this submission.

4. **The Supplemental information section: the data presented in this section is more than the data you presented in the original paper, so please add these data to the original results not as a supplement**

We rearranged the calibration and growth figures from the supplemental section, please find these figures with this submission.

5. **The statistical data and figure you illustrate in the supplementary information should be translated and represented in the paper result and discussion not as a Supplemental information.**

We rearranged the statistical figures from the supplemental section, please find these figures with this submission.

**Reviewer 2:**

1. **General comment: This is an in-vitro method to evaluate wound dressing. But this work should have included an in-vivo animal model to actually evaluate the wound dressing in presence of biological fluids such as pus (which results from bacterial infections). In addition, the absorption of silver ion through the skin is another factor that wasn’t taken into consideration. That’s why I strongly suggest performing an in-vivo animal model with wound infection.**

We appreciate the significance of the experiments recommended by the reviewers regarding in vivo testing and silver ion release measurements from wound dressings. These experiments would benefit the scope of this work in the future to enhance our knowledge of product efficacy. To provide clarity on the present work, we would appreciate sharing the following:

At present, regulatory review and approval of ion-containing wound dressings to test anti-microbial properties for regulatory ‘indications of use’ in the U.S. require in vitro testing using the standard AATCC-100. Uniquely, the goal of this work was to compare the regulatory standard accepted by FDA, AATCC-100 for microbial viability testing with methods, such as qRT-PCR which can capture viable but non-culturable organisms (dormant organisms, though viable, which may not actively grow using agarose plating methods).  Molecular methods (such as qRT-PCR) allow us to measure non-culturable organisms, that otherwise could be considered non-viable if measured using plating techniques as a sole method. Using qRT-PCR we measured for gene transcription (mRNA synthesis) specific to 16sRNA – mRNA to this gene is produced by viable bacteria and can be used to measure for small concentrations of organisms that may not be detectable through serial dilution and plating. Therefore, the focus of our work was markedly to compare methods of detection for organisms exposed to silver ion from the wound dressings.

Related to this point of methods comparison, the current standard has a 24h test period—however some metal-ion containing wound dressings may indicate longer periods of use.  Therefore, the second goal of this work was to measure microbial viability at time points greater than 24h, to measure survival or proliferation of surviving bacteria. Additionally, this test provided us with the opportunity to examine if agar-plating methods captured culturable organisms and compare these results with viable organisms captured using qRT-PCR.  Again, this provides us with the opportunity to test currently regulatory standards (specifically in this case the period of incubation and testing (24h)) with longer test periods.

The measurement of silver ion will be a valuable test to incorporate into future iterations of this research. At present, this study focused on testing these methods that could help address products that contain antimicrobial metal ions, in addition to Ag, this includes Au, Cu, Zn, Fe, and Ti [1]. In future studies in addition to Ag concentrations we may measure other ions over extended test periods to identify what the levels of ions are within the dressing. At present, this study, though we tested Ag ions, is not uniquely focused on silver-ion containing dressings but is used as an example test product to assist with evaluating our microbial viability test method, using qRT-PCR.

Similarly, we value the experimental data and work by Lakshimipriya Sethuram et al [2], which describes how these products perform on a systematic level that includes biology important to the immune system (innate and adaptive), the contributions of skin organisms and inter/intra kingdom communication which contribute to a dynamic wound environment for product testing. As our goal was to evaluate current standards used specifically for in vitro testing, for regulatory review and product clearance, at this stage our work is not focused on animal testing as it would be external to our regulatory science research goals. We value the insights through academia and other research bodies to help with our collective understanding of product efficacy as provided through:

1. Wang, H., et al., *Application of metal-based biomaterials in wound repair.* Engineered Regeneration, 2021. **2**: p. 137-153.

2. Sethuram, L., et al., *Effects and formulation of silver nanoscaffolds on cytotoxicity dependent ion release kinetics towards enhanced excision wound healing patterns in Wistar albino rats.* RSC Adv, 2019. **9**(61): p. 35677-35694.

2. **The whole document should be represented as numbered lines to facilitate the reviewing process.**

We added the numbered lines to the document.

3. **Title It should include the word “in-vitro” to be more precise.**

We changed our title to be: Modified in-vitro AATCC-100 procedure to measure viable bacteria from wound dressings

4. **Abstract Is very vague and subjective without mentioning any of the results. I suggest adding major findings (with numbers) in addition to a conclusion sentence.**

We included the following statement in the abstract:

“These results showed antimicrobial properties of wound dressings diminished as incubation days are increased for both methods from day 1 PCR result of 4.31 ± 0.54 and day 1 plating result of 6.31 ± 3.04 to day 3 PCR result of 1.22 ± 0.97 and day 3 plating result of 5.89 ± 2.41. These results show that data from qRT-PCR generally produced lower standard deviation than that of culture methods, hence shown to be more precise. Complementary parallel analysis of samples using both methods better characterized antimicrobial properties of the tested samples.”

5. **Introduction It should be written as a continuous section without sub-headings. The paragraphs must show a reasonable flow as you present the whole subject. Each paragraph represents an idea “but without a heading”. By the end of the intro the reader should know the aim of the study.**

We updated the introduction by making it continuous and rearranging details in increasing technicality.

For the aim of the study, we included the following statement:

“To this end, our objective was to assess this accepted protocol with quantitative real-time polymerase chain reaction (qRT-PCR), to measure time dependent antimicrobial efficacy of wound dressings, and to examine for potential viable bacteria but non-culturable bacteria as compared with traditional plating methods. This investigation features an extended version of this method, with 3- and 7-day incubation with addition of simulated wound fluids. This study describes a protocol that could expand upon AATCC-100, featuring multiple time points for testing that are representative of the product’s ‘Indications of Use’. This latter assay can track changes in antimicrobial properties of dressings over a longer duration of incubation.”

6. **Please number each heading and sub-headings with multilevel list headings.**

We followed the guideline to apply the multilevel list headings.

7. **Please show the positive and negative controls for each experiment and explain the statistical evaluation for each exp.**

We replaced the term previously used as “positive and negative treatment” with “positive and negative control” to better illustrate our findings.

8. **I suggest that you perform another experiment showing the release of silver ion from the wound dressing in-vitro over time and in simulated in-vivo experiments.**

Similar to our response earlier, we value both the experimental data and work by Lakshimipriya Sethuram et al [2] and Kristina Nešporová et al [3]. The former studies silver ion release kinetics in in vivo conditions while the latter studies in vitro conditions. As our goal was to evaluate current standards used specifically for in vitro testing, for regulatory review and product clearance, at this time our work is not focused on animal testing as it would be external to the scope of our regulatory science research. We value the insights through academia and other research bodies to help with our collective understanding of product efficacy and provided through:

3. Nešporová K, Pavlík V, Šafránková B, Vágnerová H, Odráška P, Žídek O, et al.. *Effects of wound dressings containing silver on skin and immune cells.* Scientific Reports. 2020;10. doi:10.1038/s41598-020-72249-3.

9. **Also, the concentration of silver ion in the skin and spleen of the animal in the in-vivo animal model should be measured to evaluate the actual release.**

Similarly, we value the experimental data and work by Lakshimipriya Sethuram et al [2], which histopathologically evaluated skin samples for silver ion concentration. As our goal was to evaluate current standards used specifically for in vitro testing, for regulatory review and product clearance, our research was not focused on animal testing as it would be external to the scope of our regulatory science. We value the insights through academia and other research bodies to help with our collective understanding of product efficacy and provided through.

10. **As it was mentioned earlier, the culture technique was evaluated as “log reduction”, so I suggest that the growth curve for both S. aureus and P. aeruginosa represented as “Log CFU/ml vs time” instead of “CFU/mL”.**

The purpose of the bacteria growth characterization curve is not only to show the expected logarithmic bacteria growth pattern but also to fit to the similarly logarithmic time vs OD_600_ graph to make a calibration curve with high R squared value (supp fig 7 and 8). A time vs log CFU / mL may present a linear trend, yet it would distort the calibration curves thereof.

11. **Figure S7: the data should have been analyzed by either: One-way ANOVA (if you compare each factor in a single day) followed by post test and multiple comparisons or TWO-way ANOVA (if you compare each factor among the three days) followed by post test for corrections and multiple comparisons.**

Thank you for your valuable feedback. Regarding the calibration curve in Fig S7, which uses the abline fitting, the intention is to depict the relationship between two variables represented by discrete data points. We understand the importance of selecting appropriate statistical analyses, and in this context, ANOVA might not be the most suitable choice given it evaluates differences in means across multiple groups. Instead, for this specific analysis, we relied on the standard deviation from the 24h incubation combined with the plate-count method to provide a more precise assessment. We have also revisited the log reduction analysis of microbial viability/culturability, ensuring that we use a suitable statistical approach. We hope this clarifies our methodology.

12. **All the figures showing more than 2 factors should have been analyzed by one-way ANOVA followed by a post test for corrections and multiple comparisons.**

Analysis Approach We concur with your suggestion regarding the selection of statistical tests. Given our intention to compare gene expressions across the ‘method’ and ‘day’ factors into *P. aeruginosa* and *S. aureus* respectively, we therefore employed a two-way ANOVA.

Post-hoc pairwise Comparisons Analysis: We'd like to clarify that we had already conducted a post-hoc pairwise comparisons analysis following the two-way ANOVA using the Tukey's HSD test. This was incorporated in the original version to ascertain specific differences among the levels of the factors. We have ensured that this is emphasized more clearly in our revised manuscript.

Clarification on Statistical Analysis: To ensure clarity and consistency in our statistical analyses, for any other figures presenting more than one independent factor, we have employed the two-way ANOVA approach and subsequently performed a post-hoc pairwise comparisons analysis to account for multiple comparisons.

Updates to Figures and Legends: In response to your feedback, we have updated the figures and legends. The plots have been revised with increased resolution, and we have updated the markers of statistical significance to ensure clarity.

13. **The first paragraph has no references, so please add recent references (2022 or 2023) to any sentence that is not your actual findings.**

We included some recent references to the first paragraph of the discussion section.

Akyildiz, H.I., B.A. Yilmaz, and S. Diler, *Antibacterial Activity of Photodeposited Ag Nanoparticles on Cotton Fibers Enabled by Atomic Layer Deposition.* Fibers and Polymers, 2022. **23**(10): p. 2769-2779.

14. **In other paragraphs please add recent references and mention recent studies.
    Conclusion I suggest you add a couple of sentences indicating your future directions.
    References. Please update the references list with more recent papers (2022 or 2023).**

We included some recent references to the manuscript.

Ahmed, T., R.T. Ogulata, and O. Gülnaz, *Excellency of Calendula arvensis in AgNPs Synthesis for Cotton Fabrics in Terms of Shape Diversity and Ant*ibacterial Wash Fastness. Fibers and Polymers, 2023.

Rybka, M., L. Mazurek, and M. Konop, *Beneficial Effect of Wound Dressings Containing Silver and Silver Nanoparticles in Wound Healing-From Experimental Studies to Clinical Practice.* Life (Basel), 2022. **13**(1).

Yuan, Y., et al., *Nano-silver functionalized polysaccharides as a platform for wound dressings: A review.* Int J Biol Macromol, 2022. **194**: p. 644-653.

Heydari Foroushani, P., et al., *Curcumin Sustained Release with a Hybrid Chitosan-Silk Fibroin Nanofiber Containing Silver Nanoparticles as a Novel Highly Efficient Antibacterial Wound Dressing.* Nanomaterials (Basel), 2022. **12**(19).
